# Higher dietary live microbe intake is linked to reduced risk of metabolic syndrome and mortality: a cross-sectional and longitudinal study

**DOI:** 10.3389/fnut.2025.1592969

**Published:** 2025-04-29

**Authors:** Shan Huang, Haixia Xiao, Huanshun Xiao, Lu Liu, Shuangming Cai

**Affiliations:** ^1^Department of MICU, Guangdong Women and Children Hospital, Guangzhou, China; ^2^Department of Obstetrics, Guangdong Women and Children Hospital, Guangzhou, China; ^3^Department of Internal Medicine, Guangdong Women and Children Hospital, Guangzhou, China

**Keywords:** metabolic syndrome, dietary live microbe, all-cause mortality, cardiovascular mortality, NHANES

## Abstract

**Background:**

The association between dietary live microbe intake and metabolic syndrome (MetS) prevalence, as well as its impact on all-cause and cardiovascular disease (CVD) mortality in MetS patients, remains underexplored.

**Methods:**

A total of 38,462 individuals from the National Health and Nutrition Examination Survey (NHANES) 1999–2018 were analyzed. Based on the live microbial level of the consumed foods, participants were divided into three dietary live microbe intake groups: low, medium, and high. Foods with medium and high live microbe content were aggregated into a medium-high consumption category. MetS was defined based on NCEP-ATP III criteria. Survey-weighted logistic regression assessed the cross-sectional association with MetS prevalence, while Cox proportional hazards models evaluated mortality risks in 12,432 individuals with MetS, among whom 2,641 all-cause and 901 CVD deaths occurred.

**Results:**

Higher dietary live microbe intake was significantly associated with lower odds of MetS. Compared to the low intake group, participants in the high intake group had a 12% lower risk of MetS in the fully adjusted model (OR: 0.88; 95% CI: 0.80–0.97; *p* = 0.01). Among MetS components, significant inverse associations were observed for low HDL-C, elevated TG, and elevated BP. Participants with high dietary live microbe intake demonstrated a significantly lower risk of all-cause mortality (HR: 0.85; 95% CI: 0.77–0.94; *p* = 0.002) and CVD-specific mortality (HR: 0.71; 95% CI: 0.55–0.92; *p* = 0.001) compared to the low intake group. Kaplan–Meier survival curves revealed better survival probabilities in individuals with medium and high intake levels, with significant differences across groups (log-rank *p* < 0.005). Quantitatively, each 100g increase in MedHi foods was associated with 6% lower all-cause mortality (HR: 0.94; 95% CI: 0.90–0.99; *p* = 0.01) and 8% lower CVD mortality (HR: 0.92; 95% CI: 0.84–1.00; *p* = 0.05).

**Conclusion:**

Dietary live microbe intake is inversely associated with the prevalence of MetS and its key components, particularly low HDL-C, elevated TG, and elevated BP. In individuals with MetS, higher live microbe intake is associated with reduced all-cause and CVD-specific mortality. These findings suggest that dietary live microbes are a promising modifiable factor for MetS prevention and management, as well as for improving long-term survival outcomes.

## 1 Introduction

Metabolic syndrome (MetS), a cluster of metabolic disorders characterized by elevated fasting glucose, hypertension, central obesity, dyslipidemia, and decreased high-density lipoprotein cholesterol, has emerged as a major global public health challenge (1). The prevalence of MetS has increased dramatically over the past decades, with current estimates indicating that approximately one-third of the U.S. population is affected, rising from 25.3% in 1988 to 34.7% in 2016 (2). This metabolic disorder has garnered substantial attention due to its strong association with increased risks of cardiovascular disease, type 2 diabetes, stroke, and all-cause mortality (3). Meta-analyses have revealed that individuals with MetS face a two-fold higher risk of cardiovascular events and a 1.5-fold increase in all-cause mortality compared to those without MetS (4, 5). Given the significant impact of MetS on public health and its rising prevalence in the context of global aging, identifying effective strategies to prevent and manage MetS has become increasingly crucial (6).

Dietary factors have long been recognized as pivotal contributors to metabolic health. In recent years, attention has been focused on the potential role of dietary live microbes—microorganisms naturally present in foods such as fermented products, fresh produce, and probiotic supplements—in influencing metabolic and overall health (7, 8). Modern food processing and preservation techniques, while improving food safety, have inadvertently reduced exposure to beneficial microbes, raising concerns about their impact on metabolic homeostasis (9, 10). The “old friends hypothesis” suggests that exposure to commensal or non-harmful microbes serves as a beneficial immune stimulant, potentially affecting metabolic regulation (11, 12). When consumed as part of the diet, these live microbes can reach the gut and colonize this compartment, interacting with resident microbes and potentially exerting positive effects on host metabolism (12, 13).

Sanders et al. have assessed the number of live microbes that are consumed in the diet and categorized all foods into low (<10^4 CFU/g), medium (10^4–10^7 CFU/g), and high (>10^7 CFU/g) levels of live microbes (14). This classification enables systematic investigation of the relationship between dietary live microbe intake and health outcomes using population-based data. They also demonstrated that diets rich in live microbes were associated with improved health outcomes, such as reduced body mass index (BMI), blood pressure, lipid levels, glucose and insulin levels, and decreased inflammation (15). Additionally, several studies have indicated that live microbes could offer protection against specific illnesses, such as cardiovascular disease (16), abdominal aortic calcification (17), sarcopenia (18), chronic constipation (19), and depression (20). However, despite the growing evidence supporting the role of dietary live microbes in metabolic health, no studies have specifically examined their relationship with MetS as a composite condition. Furthermore, the potential impact of dietary live microbes on mortality outcomes among individuals with MetS has not been comprehensively examined.

To address these knowledge gaps, we conducted a comprehensive cross-sectional and longitudinal study to investigate the association between dietary live microbe intake and both MetS prevalence and mortality risk. Our study utilized a large population-based dataset to examine whether higher consumption of foods containing live microbes is associated with reduced risk of MetS and improved survival outcomes. This research provides novel insights into the potential role of dietary live microbes in metabolic health and mortality risk, with important implications for public health recommendations and clinical practice. Understanding these relationships is crucial for developing evidence-based dietary strategies to combat the rising prevalence of MetS and its associated mortality burden.

## 2 Materials and methods

### 2.1 Study population and design

The NHANES is a large, multistage, comprehensive survey of civilian, noninstitutionalized US populations that is conducted by the CDC’s National Center for Health Statistics (NCHS, Hyattsville, MD, USA). A representative sample of the US population was sampled using a stratified, multistage cluster probability sampling design (21, 22). All data are publicly available and can be accessed at the NHANES^1^ website. NHANES has acquired written informed consent from each participant. Approval was obtained from the Research Ethics Review Board of the National Center for Health Statistics. Therefore, no additional ethical approval was needed for this analysis.

**Figure 1:**
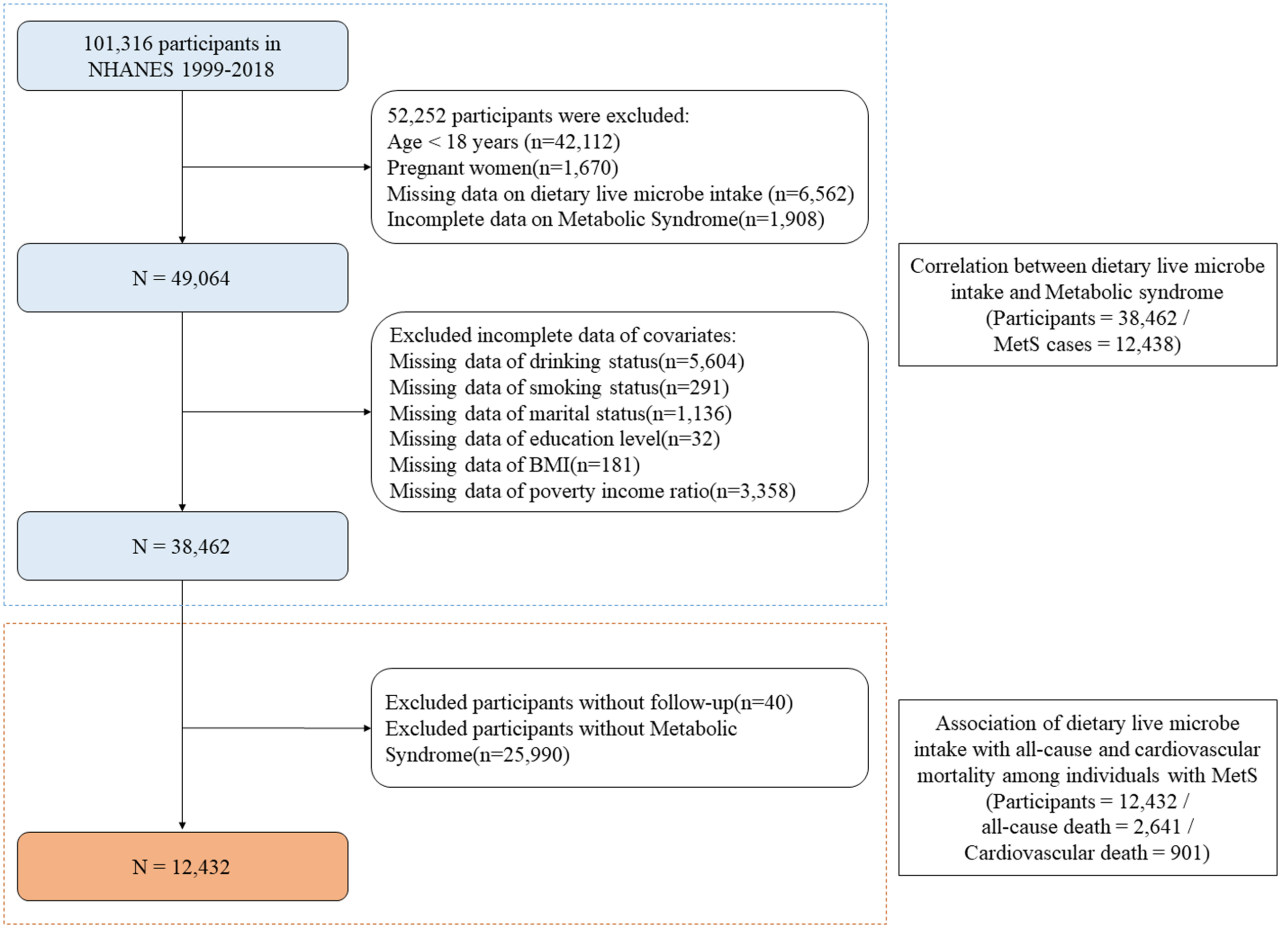
Flowchart of the sample selection from the National Health and Nutrition Examination Survey 1999 to 2018. MetS, Metabolic syndrome; BMI, Body mass index; NHANES, National Health and Nutrition Examination Survey.

Part I: We obtained data from NHANES cycles 1999–2018, which initially included 101,316 participants. After applying exclusion criteria (age <18 years [*n* = 42,112], pregnant women [*n* = 1,670], missing data on dietary live microbe intake [*n* = 6,562], and incomplete data on Metabolic Syndrome [*n* = 1,908]), 49,064 participants remained. Further exclusions were made for incomplete covariate data including drinking status (*n* = 5,604), smoking status (*n* = 291), marital status (*n* = 1,136), education level (*n* = 32), BMI (*n* = 181), and poverty income ratio (*n* = 3,358). Finally, 38,462 participants (including 12,438 MetS cases) were selected. The cross-sectional association between dietary live microbe intake and MetS was explored (Figure 1). In our cross-sectional analysis, ‘baseline’ refers to the single NHANES examination cycle when all measurements were collected. MetS components were assessed during the mobile examination center visit, while dietary live microbe intake was evaluated through a 24-h dietary recall conducted either on the same day or within a few days of the examination.

Part II: For the mortality analysis, after excluding 40 participants who were lost to follow-up from the MetS cases, 12,432 participants with MetS were included. Among these participants, 2,641 all-cause deaths and 901 cardiovascular deaths were recorded. The longitudinal relationship between dietary live microbe intake and mortality was investigated among individuals with MetS (Figure 1).

### 2.2 Measurement of dietary live microbial intakes

The dietary data in this study were obtained through an in-person 24-h dietary recall using the What We Eat in America questionnaire, which utilized the automated multiple-pass method developed by the US Department of Agriculture (USDA). The assessment of food intake containing microbes and fermented foods was conducted using the methodology developed by Sanders et al. (14). In summary, a group of experts, relying on values reported in the primary literature, estimated the levels of live microbes (CFU/g) for 9,388 food codes across 48 subgroups in the NHANES database. The estimated content of live microbes in the foods was used to classify them into three categories: low (< 10^4 CFU/g foods), medium (10^4–10^7 CFU/g foods), and high (> 10^7 CFU/g foods). Participants were divided into three groups based on the concentration of live microbes in the foods they consumed, not the quantity of food consumed: the low dietary live microbe intake group (consuming only low-level foods, typically pasteurized foods), the medium dietary live microbe intake group (consuming medium but not high-level foods, typically unpeeled fresh fruits and vegetables), and the high dietary live microbe intake group (consuming any high-level foods, typically unpasteurized fermented foods and probiotic supplements). Additionally, we aggregated the amounts of medium-level and high-level foods consumed per person (in 100 grams) and defined this as the MedHi consumption category as in previous study (23). We employed two complementary approaches to evaluate dietary live microbe intake: (1) a categorical classification based on the microbe concentration in consumed foods, which captures qualitative differences in dietary patterns; and (2) a continuous measure (MedHi) quantifying the amount of medium-high microbe foods consumed, which reflects the dose–response relationship. These dual approaches provide complementary insights: the three-category classification reflects typical dietary patterns for practical clinical interpretation, while the MedHi consumption analysis as both continuous and categorical variables enables exploration of dose–response relationships that might not be captured by the three-level classification alone.

### 2.3 Assessment of outcomes

#### 2.3.1 Part I: assessment of MetS and its components

According to the Adult Treatment Program III of the National Cholesterol Education Program (NCEP-ATP III) criteria (24), MetS was diagnosed if it includes at least three components: (1) Elevated fasting plasma glucose (FPG) ≥ 6.1 mmol/L (110 mg/dL); (2) Low high-density lipoprotein cholesterol (HDL-C) < 1.29 mmol/L (50 mg/dL) in women, < 1.03 mmol/L (40 mg/dL) in men; (3) Elevated serum triglycerides (TG) > 1.69 mmol/L (150 mg/dL); (4) Elevated waist circumference (WC) > 88 cm in women or >102 cm in men; (5) Elevated blood pressure (BP) > 130/85 mmHg.

#### 2.3.2 Part II: assessment of mortality

A public-use linked mortality file for the NHANES 1999 to 2018 has been generated by the National Center for Health Statistics based on data from the National Death Index. The primary outcomes were death attributable to any cause and CVD. The International Statistical Classification of Diseases and Related Health Problems, Tenth Revision (ICD-10) is used to document the underlying causes of death. CVD-specific mortality was defined as ICD-10 codes I00-I09, I11, I13, I20-I51, and I60-I69. The duration of follow-up was defined as the interval in months from the date of interview to the date of death or to December 31, 2019.

### 2.4 Assessment of covariates

Demographic and socioeconomic information was collected via questionnaires, including age, sex (male or female), and race (categorized as Mexican American, other Hispanic, non-Hispanic white, non-Hispanic black, or other race). Marital status was classified as married/cohabit, divorced/separated/widowed, and never married, and the poverty income ratio (PIR) was categorized as <1.3, 1.3–3.5, or >3.5. Education level was classified as lower than high school, high school, and above high school. Anthropometric and lifestyle factors were also assessed. Body mass index (BMI) was calculated as weight in kilograms divided by height in meters squared and classified as <25, 25–30, or >30 kg/m2. Food calorie intake was considered continuous variables. Smoking status was classified as current, former, and never smoker. Drinking status was categorized as now, former, and never. The total time of physical activity (PA) was self-reported by the participants by using the Global Physical Activity Questionnaire (GPAQ) which was created by the World Health Organization (WHO). PA level was categorized into three levels: inactive (0 MET-min/week), insufficiently (1–599 MET-min/week), and sufficiently (≥600 METmin/week), based on the definition of American Physical Activity Guideline (25).

### 2.5 Statistical analysis

Following NHANES analytic recommendations, the complex sampling design and appropriate mobile examination center (MEC) sample weights were applied in all analyses. Specifically, for the 1999–2002 cycle, weights were calculated as weight = 2/10 × wtmec4yr; for the 2003–2018 cycle, weights were calculated as weight = 1/10 × wtmec2yr. We selected MEC weights instead of dietary weights (‘wtdr1d’ or ‘wtdr2d’) because our analyses involved both dietary intake and physical/laboratory measurements, such as metabolic syndrome components and mortality follow-up, which are best represented using MEC weights. The weighted analyses were performed with the R package “survey.” Continuous variables are presented as means with standard errors (SE), and categorical variables are shown as proportions. Differences between groups were evaluated using weighted linear regression for continuous variables or weighted chi-square testing for categorical variables.

Survey-weighted multiple logistic regression analysis was used to evaluate the relationships between dietary live microbe intake and the prevalence of MetS, and three models were constructed. There was no covariate adjustment in model 1. Age, sex, and race-related modifications were made to model 2. Model 3 was adjusted for the Model 2 variables plus education level, marital status, PIR, BMI, smoking status, drinking status, and physical activity. Meanwhile, survey-weighted multiple logistic regression analyses were also performed to clarify the association between dietary live microbe intake and the 5 individual MetS component prevalences (elevated FPG, low HDL-C, elevated TG, elevated WC, and elevated BP). The gram of foods containing medium-high dietary live microbe (MedHi) was used as both continuous and categorical variables (quartiles) to explore the relationships between MedHi and MetS or mortality. Restricted cubic spline (RCS) regression was applied to allow for visual and statistical assessment of nonlinear associations between MedHi and MetS and its components. Stratification analysis was implemented to investigate whether the association between dietary live microbe intake and MetS was sustained across the different subgroups classified using age, sex, race, marital status, education level, smoking status, drinking status, BMI, PIR, and PA. These stratification variables were also considered as pre-specified possible impact modifiers. To test for heterogeneity of associations between subgroups, an interaction term was also introduced.

We performed weighted Kaplan–Meier (KM) curves with the log-rank tests for cumulative survival differences across different dietary live microbe intake groups. Survey-weighted Cox proportional hazards regression analyses were used to examine the relationship between dietary live microbe intake and all causes and CVD mortality in the multivariable model, where models were created consistent with the above (survey-weighted multivariable logistic regression analyses). We used all statistical analyses using R software (version 4.3.3). *p* values <0.05 from two-tailed tests were considered statistically significant.

## 3 Results

### 3.1 Baseline characteristics of study participants by dietary live microbe intake

Table 1 presents the clinical characteristics of 38,462 individuals categorized by different dietary live microbe intake groups (Low, Medium, and High). The average age of the study population was 46.87 ± 0.20 years, with a gender distribution of 50.57% females and 49.43% males, and 29.78% of the participants were diagnosed with MetS. After grouping the dietary live microbe intake, the low intake group represented 35.63% of the participants, the medium intake group represented 42.88%, and the high intake group represented 21.49%.

**Table 1:** The clinical characteristics of the study population according to the different dietary live microbe intake groups.

Variable	Total	Dietary live microbe intake group			*p* value
		Low	Medium	High	
No. of participants (%)		13,682 (35.63)	16,492 (42.88)	8,288 (21.49)	
Age (years)	46.87 (0.20)	45.05 (0.22)	48.45 (0.26)	46.60 (0.28)	< 0.0001
Age group, *n* (%)					< 0.0001
<39	12,163 (34.97)	4,684 (39.09)	4,664 (31.50)	2,815 (35.37)	
<59	12,902 (39.00)	4,523 (38.51)	5,547 (39.49)	2,832 (38.84)	
60+	13,397 (26.03)	4,475 (22.39)	6,281 (29.01)	2,641 (25.79)	
Sex, *n* (%)					< 0.0001
Male	19,407 (49.43)	7,404 (54.07)	8,234 (48.35)	3,769 (45.32)	
Female	19,055 (50.57)	6,278 (45.93)	8,258 (51.65)	4,519 (54.68)	
Race, *n* (%)					< 0.0001
Non-Hispanic White	18,014 (70.82)	5,664 (65.01)	7,709 (70.71)	4,641 (78.35)	
Mexican American	6,493 (7.59)	1988 (7.16)	3,326 (9.10)	1,179 (5.71)	
Non-Hispanic Black	7,834 (10.33)	3,869 (15.53)	2,894 (9.07)	1,071 (5.80)	
Other Hispanic	2,983 (5.14)	1,066 (5.77)	1,240 (4.97)	677 (4.60)	
Other race	3,138 (6.12)	1,095 (6.53)	1,323 (6.15)	720 (5.54)	
Marital status, *n* (%)					< 0.0001
Married/cohabit	23,326 (64.39)	7,613 (59.19)	10,401 (66.32)	5,312 (67.83)	
Never married	6,684 (17.42)	2,760 (20.61)	2,516 (15.50)	1,408 (16.49)	
Divorced/separated/widowed	8,452 (18.19)	3,309 (20.20)	3,575 (18.18)	1,568 (15.68)	
Education level, *n* (%)					< 0.0001
Lower than high school	9,634 (15.82)	4,078 (21.05)	4,201 (15.63)	1,355 (9.53)	
High school	8,980 (24.07)	3,596 (28.47)	3,750 (23.40)	1,634 (19.58)	
Above high school	19,848 (60.11)	6,008 (50.47)	8,541 (60.96)	5,299 (70.88)	
PIR, *n* (%)					< 0.0001
<1.3	11,447 (20.38)	4,982 (27.08)	4,586 (18.79)	1879 (14.50)	
1.3–3.5	14,665 (35.73)	5,371 (38.81)	6,383 (35.62)	2,911 (32.02)	
>3.5	12,350 (43.89)	3,329 (34.10)	5,523 (45.59)	3,498 (53.48)	
BMI (kg/m^2^)	28.76 (0.07)	29.23 (0.09)	28.62 (0.08)	28.37 (0.11)	< 0.0001
BMI group					< 0.0001
<25	11,358 (31.13)	3,941 (29.48)	4,819 (31.26)	2,598 (32.99)	
25–30	13,047 (33.28)	4,391 (31.35)	5,791 (34.13)	2,865 (34.35)	
30+	14,057 (35.59)	5,350 (39.17)	5,882 (34.61)	2,825 (32.66)	
Smoking status, *n* (%)					< 0.0001
Never	20,493 (53.35)	6,768 (48.52)	9,014 (54.68)	4,711 (57.32)	
Former	9,722 (25.08)	3,095 (21.90)	4,469 (26.89)	2,158 (26.16)	
Current	8,247 (21.57)	3,819 (29.58)	3,009 (18.43)	1,419 (16.52)	
Drinking status, *n* (%)					< 0.0001
Never	5,212 (10.75)	1942 (11.39)	2,336 (11.57)	934 (8.59)	
Former	6,694 (14.24)	2,703 (16.53)	2,805 (13.92)	1,186 (11.86)	
Now	26,556 (75.02)	9,037 (72.08)	11,351 (74.50)	6,168 (79.55)	
Energy intake (kcal/day)	2137.55 (6.88)	2073.92 (10.80)	2138.01 (8.99)	2214.94 (12.88)	< 0.0001
PA, *n* (%)					< 0.0001
Inactive	9,952 (20.89)	4,052 (24.71)	4,153 (20.41)	1747 (16.85)	
Insufficiently	9,461 (26.33)	3,201 (25.99)	4,212 (26.84)	2048 (25.92)	
Sufficiently	19,049 (52.78)	6,429 (49.30)	8,127 (52.75)	4,493 (57.23)	
MetS, *n* (%)					< 0.0001
No	26,024 (70.22)	9,149 (68.54)	11,003 (69.64)	5,872 (73.26)	
Yes	12,438 (29.78)	4,533 (31.46)	5,489 (30.36)	2,416 (26.74)	
Elevated FPG, *n* (%)					0.12
No	27,691 (75.39)	9,824 (75.35)	11,677 (74.81)	6,190 (76.36)	
Yes	10,771 (24.61)	3,858 (24.65)	4,815 (25.19)	2098 (23.64)	
Low HDL-C, *n* (%)					< 0.0001
No	26,887 (70.63)	9,246 (66.35)	11,616 (71.43)	6,025 (74.74)	
Yes	11,575 (29.37)	4,436 (33.65)	4,876 (28.57)	2,263 (25.26)	
Elevated TG, *n* (%)					< 0.0001
No	25,371 (66.27)	9,044 (64.94)	10,699 (65.78)	5,628 (68.74)	
Yes	13,091 (33.73)	4,638 (35.06)	5,793 (34.22)	2,660 (31.26)	
Elevated WC, *n* (%)					0.003
No	17,007 (45.30)	5,947 (43.84)	7,260 (45.29)	3,800 (47.17)	
Yes	21,455 (54.70)	7,735 (56.16)	9,232 (54.71)	4,488 (52.83)	
Elevated BP, *n* (%)					< 0.0001
No	23,768 (66.57)	8,414 (66.88)	9,892 (64.42)	5,462 (69.66)	
Yes	14,694 (33.43)	5,268 (33.12)	6,600 (35.58)	2,826 (30.34)	

Weighted means (standard errors) and frequency (weighted percentages) are presented for continuous variables and categorical variables, respectively. PIR, poverty income ratio; BMI, body mass index; PA, physical activity; MetS, Metabolic syndrome; FPG, fasting plasma glucose; HDL-C, high-density lipoprotein cholesterol; TG, triglycerides; WC, waist circumference; BP, blood pressure.

The prevalence of MetS in the low, medium, and high intake groups were 31.46, 30.36, and 26.74%, respectively. Among the three dietary live microbe intake groups, statistically significant differences were found for age, sex, race, education levels, marital status, PIR, BMI, smoking status, drinking status, physical activity, and energy intake (all *p* values < 0.0001). Among MetS components, low HDL-C, elevated TG, elevated WC, and elevated BP showed statistically significant (all *p* values < 0.0001). There was no statistically significant difference in elevated fasting plasma glucose (FPG) among the groups (*p* value = 0.12).

### 3.2 Association between dietary live microbe intake and MetS

Table 2 presents the results of the multivariable logistic regression analysis examining the association between dietary live microbe intake and MetS and its components. The analysis was conducted using three models with increasing levels of adjustment for potential confounders. In the crude model without adjustments (Model 1), the high dietary live microbe intake group was significantly associated with decreased odds of MetS compared to the low intake group (OR = 0.80; 95% CI, 0.73–0.86; *p* < 0.0001). In the fully adjusted model (Model 3), there was also a significant inverse association between high dietary live microbe intake and MetS (OR = 0.88; 95% CI, 0.80–0.97; *p* = 0.01). This suggests that participants in the high intake group had a 12% lower risk of MetS compared to those in the low intake group. The trend across intake groups was also significant (*p* for trend = 0.009).

**Table 2:** Association of dietary live microbe intake with MetS and its components.

Outcome	Model 1		Model 2		Model 3	
	OR (95% CI)	*p* value	OR (95% CI)	*p* value	OR (95% CI)	*p* value
MetS						
Low	1 (Reference)		1 (Reference)		1 (Reference)	
Medium	0.95 (0.89, 1.02)	0.13	0.81 (0.76, 0.87)	<0.0001	0.94 (0.87, 1.02)	0.16
High	0.80 (0.73, 0.86)	<0.0001	0.72 (0.67, 0.79)	<0.0001	0.88 (0.80, 0.97)	0.01
*p* for trend	<0.0001		<0.0001		0.009	
Elevated FPG						
Low	1 (Reference)		1 (Reference)		1 (Reference)	
Medium	1.03 (0.96, 1.11)	0.44	0.93 (0.86, 1.01)	0.08	1.01 (0.93, 1.10)	0.81
High	0.95 (0.86, 1.04)	0.23	0.96 (0.87, 1.06)	0.41	1.07 (0.97, 1.18)	0.15
*p* for trend	0.278		0.359		0.169	
Low HDL-C						
Low	1 (Reference)		1 (Reference)		1 (Reference)	
Medium	0.79 (0.74, 0.84)	<0.0001	0.76 (0.71, 0.81)	<0.0001	0.88 (0.83, 0.95)	<0.001
High	0.67 (0.62, 0.72)	<0.0001	0.63 (0.58, 0.68)	<0.0001	0.77 (0.71, 0.84)	<0.0001
*p* for trend	<0.0001		<0.0001		<0.0001	
Elevated TG						
Low	1 (Reference)		1 (Reference)		1 (Reference)	
Medium	0.96 (0.91, 1.02)	0.20	0.88 (0.83, 0.94)	<0.0001	0.97 (0.91, 1.03)	0.29
High	0.84 (0.78, 0.91)	<0.0001	0.80 (0.74, 0.87)	<0.0001	0.90 (0.82, 0.98)	0.01
*p* for trend	<0.0001		<0.0001		0.015	
Elevated WC						
Low	1 (Reference)		1 (Reference)		1 (Reference)	
Medium	0.94 (0.89, 1.00)	0.06	0.81 (0.76, 0.86)	<0.0001	0.90 (0.81, 1.00)	0.05
High	0.87 (0.81, 0.94)	<0.0001	0.77 (0.71, 0.84)	<0.0001	0.91 (0.80, 1.04)	0.16
*p* for trend	<0.001		<0.0001		0.146	
Elevated BP						
Low	1 (Reference)		1 (Reference)		1 (Reference)	
Medium	1.11 (1.05, 1.19)	<0.001	0.93 (0.86, 1.00)	0.05	1.01 (0.93, 1.09)	0.84
High	0.88 (0.81, 0.95)	0.001	0.81 (0.74, 0.89)	<0.0001	0.90 (0.81, 0.99)	0.03
*p* for trend	0.005		<0.0001		0.038	

OR, odds ratio; CI, confidence interval; MetS, Metabolic syndrome; FPG, fasting plasma glucose; HDL-C, high-density lipoprotein cholesterol; TG, triglycerides; WC, waist circumference; BP, blood pressure; PIR, poverty income ratio; BMI, body mass index.

Model 1: There are no covariates were adjusted.

Model 2: Age, sex, and race were adjusted.

Model 3: Age, sex, race, education level, marital status, PIR, BMI, smoking status, drinking status, and physical activity were adjusted.

In addition, Table 2 also depicts the association between the dietary live microbes and the five MetS components in various models. Using multivariate regression analysis with a complex sampling design, we discovered that dietary live microbes were significantly linked with low HDL-C, elevated TG, and elevated BP, but not with FPG and WC. In particular, for HDL-C, both medium and high intake groups showed significantly lower risks compared to the low intake group (Medium: OR = 0.88; 95% CI, 0.83–0.95; *p* < 0.001; High: OR = 0.77; 95% CI, 0.71–0.84; *p* < 0.0001). The trend across groups was highly significant (*p* for trend < 0.0001). For TG, the high intake group showed a significantly lower risk (OR = 0.90; 95% CI, 0.82–0.98; *p* = 0.01). The trend across groups was significant (*p* for trend = 0.015). For BP, the high intake group showed a significantly lower risk (OR = 0.90; 95% CI, 0.81–0.99; *p* = 0.03). The trend across groups was significant (*p* for trend = 0.038). For WC, after full adjustment, the associations were no longer statistically significant, although there was a trend toward lower risk in the medium intake group (OR = 0.90; 95% CI, 0.81–1.00; *p* = 0.05). For FPG, no significant association was found in any model.

To address potential confounding by energy intake, we performed additional analyses with adjustment for dietary energy intake (Supplementary Table S1). After this adjustment, high dietary live microbe intake remained significantly associated with lower odds of MetS (OR = 0.86; 95% CI: 0.77–0.95; *p* = 0.004) compared to the low intake group in the fully adjusted model. For individual MetS components, significant inverse associations persisted for low HDL-C (OR = 0.74; 95% CI: 0.68–0.81; *p* < 0.0001), elevated TG (OR = 0.87; 95% CI: 0.79–0.95; *p* = 0.003), and elevated BP (OR = 0.90; 95% CI: 0.82–1.00; *p* = 0.05). Interestingly, after energy adjustment, we observed a significant positive association between high dietary live microbe intake and elevated FPG (OR = 1.11; 95% CI: 1.01–1.23; *p* = 0.03).

Stratified analysis revealed that the relationships between dietary live microbe intake and MetS varied across different subgroups (Table 3). The most pronounced protective effects against MetS were observed in participants aged 39–59 years (OR = 0.80; 95% CI, 0.68–0.93), those with above high school education (OR = 0.83; 95% CI, 0.73–0.95), never smokers (OR = 0.81; 95% CI, 0.70–0.94), and those with PIR > 3.5 (OR = 0.78; 95% CI, 0.66–0.91), all showing statistical significance in the high intake group compared to the low intake group. Notably, married/cohabiting individuals (OR = 0.83; 95% CI, 0.72–0.94), those who were insufficiently active (OR = 0.76; 95% CI, 0.64–0.90), and never drinkers (OR = 0.78; 95% CI, 0.62–0.98) also showed significant protective associations with high dietary live microbe intake. Interaction tests revealed significant differences in the associations between dietary live microbe intake and MetS for education level (*p* for interaction < 0.001) and PIR (*p* for interaction = 0.022), suggesting that these socioeconomic factors might modify the relationship between dietary live microbe intake and MetS.

**Table 3:** Subgroup analysis for the association between dietary live microbe intake and MetS.

Subgroup	Low OR (95% CI)	Medium OR (95% CI)	High OR (95% CI)	*p* for trend	*p* for interaction
Age					0.071
<39	1 (Reference)	0.87 (0.74, 1.01)	0.97 (0.82, 1.15)	0.574	
39–59	1 (Reference)	0.94 (0.82, 1.06)	0.80 (0.68, 0.93)	0.005	
60+	1 (Reference)	1.02 (0.89, 1.17)	0.94 (0.81, 1.10)	0.448	
Sex					0.363
Male	1 (Reference)	0.98 (0.87, 1.10)	0.90 (0.77, 1.04)	0.171	
Female	1 (Reference)	0.92 (0.81, 1.03)	0.87 (0.76, 1.00)	0.05	
Race					0.14
Non-Hispanic White	1 (Reference)	0.92 (0.82, 1.03)	0.87 (0.76, 0.99)	0.029	
Mexican American	1 (Reference)	1.01 (0.85, 1.18)	0.88 (0.71, 1.10)	0.271	
Non-Hispanic Black	1 (Reference)	1.05 (0.91, 1.21)	0.98 (0.80, 1.19)	0.925	
Other Hispanic	1 (Reference)	1.07 (0.82, 1.39)	0.90 (0.63, 1.17)	0.539	
Other Race	1 (Reference)	0.99 (0.76, 1.29)	1.13 (0.78, 1.63)	0.557	
Marital status					0.124
Married/cohabit	1 (Reference)	0.91 (0.82, 1.00)	0.83 (0.72, 0.94)	0.004	
Never married	1 (Reference)	1.12 (0.89, 1.41)	1.06 (0.79, 1.43)	0.603	
Divorced/separated/Widowed	1 (Reference)	0.96 (0.82, 1.12)	0.97 (0.79, 1.18)	0.7	
Education level					< 0.001
Less than high school	1 (Reference)	0.96 (0.84, 1.11)	0.85 (0.69, 1.04)	0.125	
High school	1 (Reference)	1.20 (1.04, 1.38)	0.98 (0.80, 1.21)	0.805	
Above high school	1 (Reference)	0.82 (0.73, 0.93)	0.83 (0.73, 0.95)	0.007	
Smoking status					0.342
Never	1 (Reference)	0.89 (0.79, 1.01)	0.81 (0.70, 0.94)	0.005	
Former	1 (Reference)	0.96 (0.82, 1.11)	0.93 (0.75, 1.14)	0.484	
Current	1 (Reference)	1.06 (0.89, 1.25)	0.98 (0.80, 1.20)	0.982	
Drinking status					0.574
Never	1 (Reference)	0.98 (0.82, 1.18)	0.78 (0.62, 0.98)	0.048	
Former	1 (Reference)	0.98 (0.85, 1.14)	0.87 (0.70, 1.08)	0.224	
Now	1 (Reference)	0.93 (0.84, 1.03)	0.90 (0.78, 1.01)	0.06	
BMI					0.414
<25	1 (Reference)	0.88 (0.70, 1.10)	0.83 (0.62, 1.10)	0.18	
25–30	1 (Reference)	0.95 (0.83, 1.08)	0.82 (0.70, 0.96)	0.015	
30+	1 (Reference)	0.95 (0.85, 1.06)	0.95 (0.83, 1.09)	0.423	
PIR					0.022
<1.3	1 (Reference)	0.98 (0.85, 1.12)	1.00 (0.84, 1.19)	0.892	
1.3–3.5	1 (Reference)	1.01 (0.90, 1.15)	0.97 (0.82, 1.13)	0.725	
>3.5	1 (Reference)	0.86 (0.74, 0.99)	0.78 (0.66, 0.91)	0.002	
PA					0.097
Inactive	1 (Reference)	1.00 (0.87, 1.16)	1.08 (0.88, 1.34)	0.505	
Insufficiently	1 (Reference)	0.94 (0.81, 1.09)	0.76 (0.64, 0.90)	0.003	
Sufficiently	1 (Reference)	0.91 (0.81, 1.03)	0.87 (0.76, 0.99)	0.035	

Each subgroup analysis was adjusted for sex, race, education level, marital status, PIR, BMI, smoking status, drinking status, and physical activity, except for the stratifying variable. OR, odds ratio; CI, confidence interval; BMI, body mass index; PIR, poverty income ratio; PA, physical activity.

### 3.3 Association between medium-high live microbe (MedHi) intake and MetS

Table 4 presents the results of multivariate regression analyses examining the association between dietary MedHi (Medium-high live microbe) food intake and MetS and its components. The analyses were conducted using three models with increasing levels of adjustment for potential confounders. In the crude model without adjustments (Model 1), continuous MedHi intake showed a significantly inverse association with MetS (OR = 0.96; 95% CI, 0.94–0.98; *p* < 0.0001), Q3 showed a significant protective effect (OR = 0.86; 95% CI, 0.79–0.95; *p* = 0.002) compared to Q1 when MedHi intake categorized into quartiles, and the trend across MedHi groups was significant (*p* for trend = 0.002). However, in the fully adjusted model (Model 3), continuous MedHi intake showed no significant association with MetS (OR = 0.98; 95% CI, 0.95–1.01; *p* = 0.11), while Q2 showed a significant protective effect (OR = 0.89; 95% CI, 0.80–0.98; *p* = 0.02) compared to Q1. As for the MetS components, significant inverse associations were observed between continuous MedHi intake and low HDL-C (OR = 0.96; 95% CI, 0.94–0.99; *p* = 0.003); both Q2 (OR = 0.88; 95% CI, 0.80–0.96; *p* = 0.01) and Q3 (OR = 0.86; 95% CI, 0.78–0.96; *p* = 0.005) showed significant protective effects compared to Q1. A borderline significant protective effect was observed between continuous MedHi intake and elevated BP (OR = 0.98; 95% CI, 0.95–1.00; *p* = 0.06). No significant associations between MedHi intake with elevated FPG, elevated TG, and elevated WC were observed in the Model 3.

**Table 4:** Association of dietary MedHi food intake with MetS and its components.

Outcome	Model 1		Model 2		Model 3	
	OR (95% CI)	*p* value	OR (95% CI)	*p* value	OR (95% CI)	*p* value
MetS						
MedHi	0.96 (0.94, 0.98)	<0.0001	0.94 (0.92, 0.96)	<0.0001	0.98 (0.95, 1.01)	0.11
MedHi group						
Q1	1 (Reference)		1 (Reference)		1 (Reference)	
Q2	0.94 (0.86, 1.03)	0.19	0.85 (0.78, 0.93)	<0.001	0.89 (0.80, 0.98)	0.02
Q3	0.86 (0.79, 0.95)	0.002	0.77 (0.70, 0.85)	<0.0001	0.91 (0.82, 1.02)	0.10
*p* for trend	0.002		<0.0001		0.101	
Elevated FPG						
MedHi	0.99 (0.97, 1.01)	0.40	0.98 (0.96, 1.00)	0.09	1.00 (0.98, 1.02)	0.90
MedHi group						
Q1	1 (Reference)		1 (Reference)		1 (Reference)	
Q2	1.00 (0.90, 1.11)	0.98	0.93 (0.84, 1.04)	0.19	0.95 (0.85, 1.06)	0.39
Q3	0.97 (0.88, 1.06)	0.48	0.89 (0.81, 0.99)	0.03	0.97 (0.87, 1.07)	0.51
*p* for trend	0.47		0.028		0.515	
Low HDL-C						
MedHi	0.93 (0.90, 0.95)	<0.0001	0.93 (0.90, 0.95)	<0.0001	0.96 (0.94, 0.99)	0.003
MedHi group						
Q1	1 (Reference)		1 (Reference)		1 (Reference)	
Q2	0.84 (0.77, 0.91)	<0.0001	0.83 (0.76, 0.91)	<0.0001	0.88 (0.80, 0.96)	0.01
Q3	0.73 (0.67, 0.80)	<0.0001	0.73 (0.67, 0.80)	<0.0001	0.86 (0.78, 0.96)	0.005
*p* for trend	<0.0001		<0.0001		0.005	
Elevated TG						
MedHi	0.97 (0.95, 1.00)	0.02	0.96 (0.94, 0.99)	0.002	0.99 (0.96, 1.01)	0.30
MedHi group						
Q1	1 (Reference)		1 (Reference)		1 (Reference)	
Q2	0.91 (0.84, 1.00)	0.05	0.88 (0.80, 0.96)	0.01	0.91 (0.83, 1.00)	0.06
Q3	0.88 (0.80, 0.96)	0.004	0.83 (0.75, 0.91)	<0.0001	0.91 (0.83, 1.01)	0.08
*p* for trend	0.004		<0.0001		0.08	
Elevated WC						
MedHi	0.95 (0.94, 0.97)	<0.0001	0.94 (0.92, 0.96)	<0.0001	1.00 (0.97, 1.04)	0.80
MedHi group						
Q1	1 (Reference)		1 (Reference)		1 (Reference)	
Q2	0.98 (0.91, 1.06)	0.58	0.89 (0.82, 0.96)	0.003	0.92 (0.82, 1.04)	0.19
Q3	0.85 (0.78, 0.93)	<0.001	0.76 (0.70, 0.83)	<0.0001	1.01 (0.87, 1.16)	0.92
*p* for trend	<0.001		<0.0001		0.874	
Elevated BP						
MedHi	0.98 (0.96, 1.00)	0.007	0.95 (0.93, 0.98)	<0.001	0.98 (0.95, 1.00)	0.06
MedHi group						
Q1	1 (Reference)		1 (Reference)		1 (Reference)	
Q2	1.11 (1.02, 1.20)	0.02	0.96 (0.87, 1.05)	0.36	0.98 (0.89, 1.08)	0.67
Q3	1.00 (0.92, 1.09)	0.98	0.83 (0.75, 0.92)	<0.001	0.91 (0.82, 1.01)	0.08
*p* for trend	0.966		<0.001		0.075	

MedHi: Medium-high live microbe as continuous variable (in 100 grams); OR, odds ratio; CI, confidence interval; MetS, Metabolic syndrome; FPG, fasting plasma glucose; HDL-C, high-density lipoprotein cholesterol; TG, triglycerides; WC, waist circumference; BP, blood pressure; PIR, poverty income ratio; BMI, body mass index.

Model 1: There are no covariates were adjusted.

Model 2: Age, sex, and race were adjusted.

Model 3: Age, sex, race, education level, marital status, PIR, BMI, smoking status, drinking status, and physical activity were adjusted.

When additionally adjusted for dietary energy intake (supplementary Table S2), continuous MedHi intake remained significantly associated with lower odds of MetS (OR = 0.97; 95% CI: 0.94–1.00; *p* = 0.03). When examined by quartiles, both Q2 (OR = 0.87; 95% CI: 0.78–0.98; *p* = 0.02) and Q3 (OR = 0.88; 95% CI: 0.79–0.99; *p* = 0.03) showed significantly lower odds of MetS compared to Q1, with a significant trend across quartiles (*p* for trend = 0.034). For MetS components, significant inverse associations persisted for low HDL-C with continuous MedHi intake (OR = 0.96; 95% CI: 0.94–0.98; *p* = 0.002) and elevated TG with quartile comparisons (Q2 OR = 0.90; 95% CI: 0.82–1.00; *p* = 0.04 and Q3 OR = 0.89; 95% CI: 0.80–0.99; *p* = 0.03).

**Figure 2:**
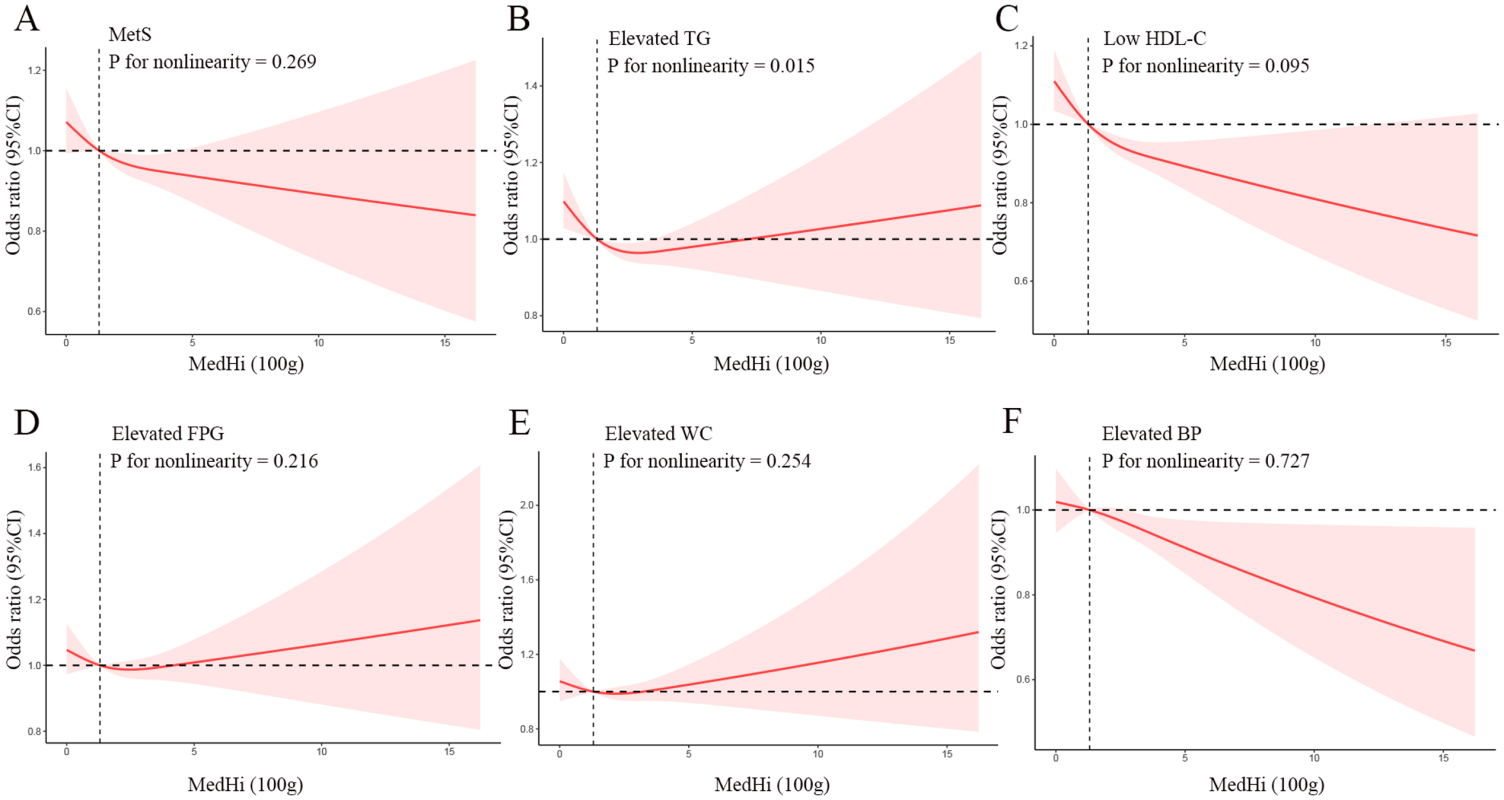
Dose–response relationships between MetS **(A)**, Elevated TG **(B)**, Low HDL-C **(C)**, Elevated FPG **(D)**, Elevated WC **(E)**, Elevated BP **(F)** and medium-high live microbe (MedHi) food intake. Model was adjusted for age, sex, race, education level, marital status, PIR, BMI, smoking status, drinking status, and physical activity. MetS, Metabolic syndrome; TG, triglycerides; HDL-C, high-density lipoprotein cholesterol; FPG, fasting plasma glucose; WC, waist circumference; BP, blood pressure; PIR, poverty income ratio; BMI, body mass index.

The restricted cubic spline (RCS) analysis revealed nonlinear associations across different outcomes in Model 3 (Figure 2). For MetS overall, there was no significant nonlinear relationship (P for nonlinearity = 0.269). While the odds ratio curve demonstrated a downward trend with increasing MedHi levels, the wide confidence intervals that consistently include 1 across most of the range indicate that this inverse trend did not reach statistical significance throughout the entire exposure range. While examining the specific components of the MetS, we found that elevated TG showed a significant nonlinear relationship (*p* for nonlinearity = 0.015) with a U-shaped curve; Low HDL-C and elevated BP displayed linear inverse relationships (*p* for nonlinearity = 0.095 and 0.727, respectively), with the risk consistently decreasing as MedHi increased; Elevated FPG and elevated WC also showed no significant nonlinearity (*p* for nonlinearity = 0.216 and 0.254, respectively), maintaining a relatively stable relationship across MedHi levels with slight fluctuations.

### 3.4 Association of dietary live microbe intake with mortality among individuals with MetS

**Figure 3:**
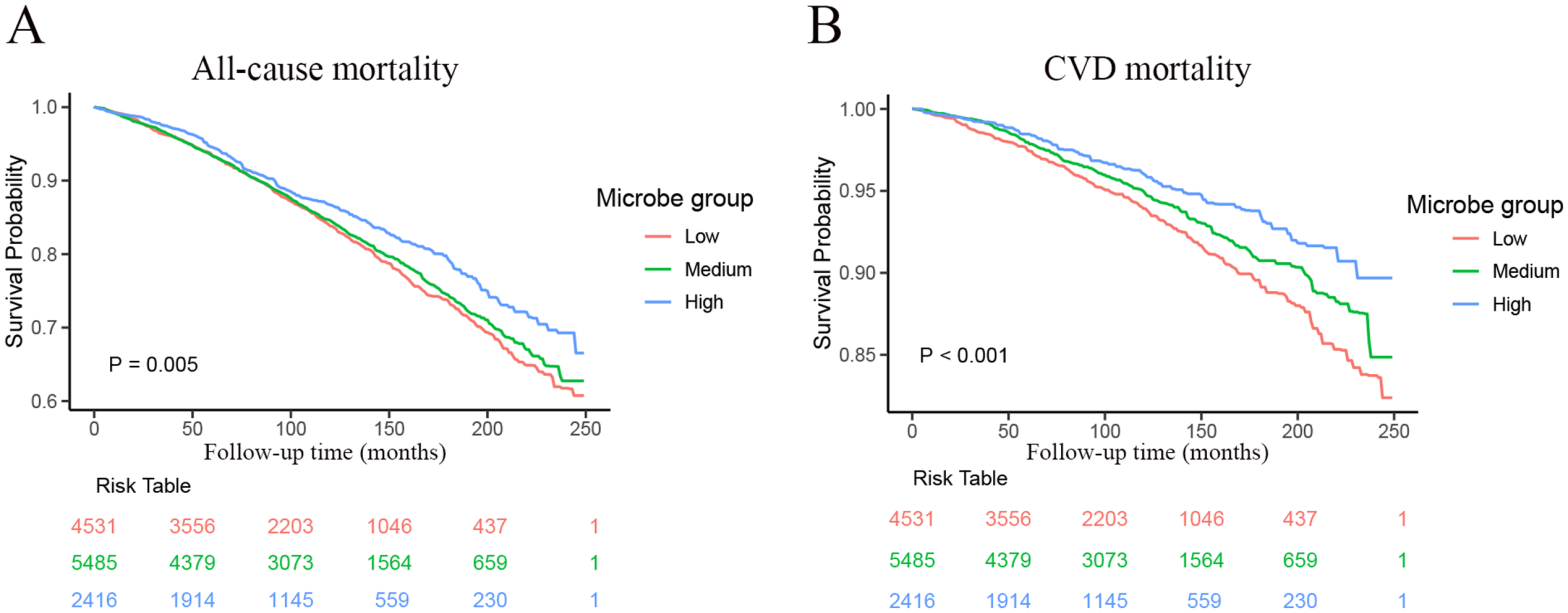
Kaplan–Meier curves were used to present the relationship of the dietary live microbe intake with all-cause and cardiovascular mortality among patients with MetS. **(A)** Refers to all-cause mortality by dietary live microbe intake groups. **(B)** Refers to cardiovascular mortality by dietary live microbe intake groups. MetS, Metabolic syndrome; CVD, cardiovascular disease.

A total of 12,432 participants with MetS were included in the analysis, with different dietary live microbe intake levels categorized as low (*n* = 4,531), medium (*n* = 5,485), and high (*n* = 2,416). During a median (IQR) follow-up of 8.5 (4.7–12.6) years, 2,641 deaths were recorded, of which 901 were attributed to CVD. The Kaplan–Meier curves depicted significant survival differences between participants with different levels of dietary live microbe intake (Figure 3). For all-cause mortality, participants with high dietary live microbe intake showed the highest survival probability, followed by those with medium intake, while the lowest survival probability was observed in the low intake group (log-rank *p* = 0.005, Figure 3A). For CVD mortality, a more pronounced separation of survival curves was observed (log-rank *p* < 0.001, Figure 3B). The high dietary live microbe intake group maintained consistently higher survival probabilities throughout the follow-up period.

Table 5 presents the associations between dietary live microbe intake and mortality outcomes among participants with MetS using different adjustment models. For all-cause mortality, the Model 1 showed that high dietary live microbe intake was associated with a 20% lower risk compared to low intake (HR = 0.80; 95% CI: 0.69–0.92; *p* = 0.002), while medium intake showed a non-significant reduction (HR = 0.95; 95% CI: 0.85–1.07; *p* = 0.43). After adjusting for basic demographic factors in Model 2, both medium and high intake groups showed identical protective effects (HR = 0.74; *p* < 0.0001 for both). However, in the fully adjusted model (Model 3), only medium intake maintained statistical significance with a 15% lower risk (HR = 0.85; 95% CI: 0.77–0.94; *p* = 0.002), while high intake showed a non-significant trend toward protection (HR = 0.90; 95% CI: 0.77–1.04; *p* = 0.15). For CVD mortality, the protective associations were more pronounced and consistent across all models. In the fully adjusted model (Model 3), both medium and high intake groups showed significant reductions in CVD mortality risk compared to low intake, with hazard ratios of 0.72 (95% CI: 0.59–0.86; *p* < 0.001) and 0.71 (95% CI: 0.55–0.92; *p* = 0.001), respectively. This represents approximately a 28–29% lower risk of CVD mortality.

**Table 5:** Multivariable-adjusted HRs and 95% CIs for dietary live microbe intake in relation to all-cause and CVD Mortality among 12,432 participants with MetS.

Outcome	Number of deaths	Model 1		Model 2		Model 3	
		HR (95% CI)	*p* value	HR (95% CI)	*p* value	HR (95% CI)	*p* value
All-cause mortality							
Low	989	1 (Reference)		1 (Reference)		1 (Reference)	
Medium	1,249	0.95 (0.85, 1.07)	0.43	0.74 (0.66, 0.82)	<0.0001	0.85 (0.77, 0.94)	0.002
High	403	0.80 (0.69, 0.92)	0.002	0.74 (0.64, 0.85)	<0.0001	0.90 (0.77, 1.04)	0.15
CVD mortality							
Low	372	1 (Reference)		1 (Reference)		1 (Reference)	
Medium	400	0.81 (0.68, 0.98)	0.03	0.62 (0.52, 0.74)	<0.0001	0.72 (0.59, 0.86)	<0.001
High	129	0.63 (0.49, 0.81)	<0.001	0.59 (0.45, 0.76)	<0.0001	0.71 (0.55, 0.92)	0.001

HR, hazard ratio; CI, confidence interval; MetS, Metabolic syndrome; CVD, cardiovascular disease; PIR, poverty income ratio; BMI, body mass index.

Model 1: There are no covariates were adjusted.

Model 2: Age, sex, and race were adjusted.

Model 3: Age, sex, race, education level, marital status, PIR, BMI, smoking status, drinking status, and physical activity were adjusted.

When further adjusted for dietary energy intake (Supplementary Table S3), the association patterns shifted slightly. For all-cause mortality, medium intake maintained a significant protective association (HR = 0.89; 95% CI: 0.80–0.98; *p* = 0.02), while the association for high intake became non-significant (HR = 0.94; 95% CI: 0.81–1.10; *p* = 0.46). For CVD mortality, both medium and high intake groups maintained significant protective associations after energy adjustment, with hazard ratios of 0.75 (95% CI: 0.62–0.91; *p* = 0.003) and 0.76 (95% CI: 0.58–0.99; *p* = 0.04), respectively. This represents approximately a 24–25% reduction in CVD mortality risk, which is slightly attenuated compared to the models without energy adjustment but remains clinically significant.

### 3.5 Association of medium-high live microbe (MedHi) intake with mortality among individuals with MetS

Table 6 presents the associations between MedHi intake (both as a continuous variable and in tertiles) and mortality outcomes among 12,432 participants with MetS across three adjustment models. For all-cause mortality, when analyzed as a continuous variable, MedHi showed consistent protective associations across all models. In the fully adjusted model (Model 3), each unit increase in MedHi was associated with a 6% lower risk of all-cause mortality (HR = 0.94; 95% CI: 0.90–0.99; *p* = 0.01). When categorized into tertiles, compared to the lowest tertile (Q1), the middle tertile (Q2) showed a borderline significant 14% reduction in mortality risk (HR = 0.86; 95% CI: 0.74–1.00; *p* = 0.05), while the highest tertile (Q3) showed a non-significant trend toward protection (HR = 0.87; 95% CI: 0.73–1.04; *p* = 0.14) after full adjustment. For CVD mortality, MedHi also demonstrated protective associations, although slightly attenuated in the fully adjusted model. As a continuous variable, each unit increase in MedHi was associated with an 8% lower risk of CVD mortality in Model 2 (HR = 0.89; 95% CI: 0.81–0.97; *p* = 0.01), which remained marginally significant after full adjustment in Model 3 (HR = 0.92; 95% CI: 0.84–1.00; *p* = 0.05). When examined by tertiles, although both Q2 and Q3 showed trends toward lower CVD mortality compared to Q1 (HR = 0.80 and 0.85, respectively), these associations did not reach statistical significance in the fully adjusted model (*p* = 0.15 and 0.32, respectively).

**Table 6:** Multivariable-adjusted HRs and 95% CIs for dietary MedHi food intake in relation to all-cause and CVD mortality among 12,432 participants with MetS.

Outcome	Number of deaths	Model 1		Model 2		Model 3	
		HR (95% CI)	*p* value	HR (95% CI)	*p* value	HR (95% CI)	*p* value
All-cause mortality							
MedHi	1,652	0.94 (0.90, 0.98)	0.004	0.91 (0.87, 0.96)	<0.001	0.94 (0.90, 0.99)	0.01
MedHi group							
Q1	584	1 (Reference)		1 (Reference)		1 (Reference)	
Q2	572	0.97 (0.84, 1.12)	0.70	0.84 (0.73, 0.96)	0.01	0.86 (0.74, 1.00)	0.05
Q3	496	0.89 (0.75, 1.06)	0.20	0.77 (0.65, 0.91)	0.002	0.87 (0.73, 1.04)	0.14
CVD mortality							
MedHi	529	0.91 (0.85, 0.99)	0.002	0.89 (0.81, 0.97)	0.01	0.92 (0.84, 1.00)	0.05
MedHi group							
Q1	183	1 (Reference)		1 (Reference)		1 (Reference)	
Q2	172	0.93 (0.69, 1.25)	0.62	0.80 (0.60, 1.07)	0.13	0.80 (0.60, 1.09)	0.15
Q3	174	0.88 (0.65, 1.19)	0.40	0.75 (0.56, 1.02)	0.07	0.85 (0.61, 1.17)	0.32

MedHi: medium-high live microbe as continuous variable (in 100 grams); MetS, Metabolic syndrome; HR, hazard ratio; CI, confidence interval; CVD, cardiovascular disease; PIR, poverty income ratio; BMI, body mass index.

Model 1: There are no covariates were adjusted.

Model 2: Age, sex, and race were adjusted.

Model 3: Age, sex, race, education level, marital status, PIR, BMI, smoking status, drinking status, and physical activity were adjusted.

The associations between MedHi intake and mortality outcomes remained consistent when additionally adjusted for dietary energy intake (Supplementary Table S4). For all-cause mortality, continuous MedHi intake maintained a significant inverse association (HR = 0.94; 95% CI: 0.90–0.98; *p* = 0.01) in the fully adjusted model including energy intake. For CVD mortality, continuous MedHi intake showed a borderline significant protective association (HR = 0.91; 95% CI: 0.83–1.00; *p* = 0.05) after adjustment for energy intake.

## 4 Discussion

This study aimed to investigate the relationship between dietary live microbe intake and MetS, as well as the association between live microbe intake and all-cause and cardiovascular disease (CVD) mortality in individuals with MetS. Our results revealed an inverse association between dietary live microbe intake and MetS prevalence, with the high intake group showing a 12% lower risk of MetS compared to the low intake group. Furthermore, medium and high levels of dietary live microbe consumption were significantly associated with reduced all-cause and cardiovascular mortality risk in MetS patients, with particularly pronounced reductions in CVD mortality of approximately 28–29%. These findings provide important evidence for live microbe intake as a potential modifiable factor for improving metabolic health and long-term survival outcomes.

Dietary live microbes refer to viable microorganisms present in fermented foods, fresh produce, and probiotic supplements (14). Fermented foods typically contain over 10^7 living microbial cells per gram, while fresh fruits and vegetables with their peels intact harbor between 10^6 and 10^8 CFU/g (26, 27). In comparison, processed foods that undergo commercial sterilization or pasteurization, along with refrigerated pasteurized products such as milk and deli meats, contain significantly fewer microorganisms, with counts below 10^4 CFU/g (28). Previous research has demonstrated that fermented foods and specific probiotics offer potential health benefits (12, 29, 30). Recent studies have assessed the contribution of dietary live microbes to various health outcomes, including prevention of necrotizing enterocolitis in infants, symptom management of functional bowel disorders, prevention of antibiotic-associated diarrhea, treatment of ulcerative colitis, and reduction of upper respiratory and gastrointestinal infections (31). Although these effects are generally modest, they tend to be beneficial for population health, suggesting the potential for incorporating adequate live microbe consumption into dietary recommendations.

Our study extends these findings by examining the associations between dietary live microbe intake and MetS and its components, as well as investigating their impact on survival outcomes in individuals with MetS. The protective association was particularly pronounced for specific MetS components - low HDL-C (23% lower risk), elevated TG (10% lower risk), and elevated BP (10% lower risk) in the high intake group. This differential pattern across MetS components warrants careful interpretation. The substantially stronger association with HDL-C compared to other components suggests that dietary live microbes may exert their most potent effects on reverse cholesterol transport and HDL metabolism pathways. This aligns with the gut microbiota can directly biotransform luminal cholesterol and convert bile salts, which affects cholesterol levels in the body (32), and mechanistic studies showing that certain bacterial strains can influence cholesterol efflux capacity and modulate expression of key proteins involved in HDL biogenesis (33). The more modest effects on TG and BP, while still significant, indicate the complexity of the effects. Interestingly, our restricted cubic spline analysis revealed that the relationship between MedHi intake and TG followed a significant U-shaped pattern (*p* for nonlinearity = 0.015), suggesting potential threshold effects where moderate consumption provides optimal benefits for triglyceride metabolism. In contrast, the linear inverse relationships observed for HDL-C and BP suggest dose-dependent benefits. These findings are consistent with a recent meta-analysis by Khalesi et al., who reported that probiotic consumption significantly reduced blood pressure (34), and with studies by Chan and Ji demonstrating improvements in lipid profiles with fermented food consumption (35, 36). Particularly intriguing is the absence of significant associations between dietary live microbe intake and elevated FPG or WC after full adjustment. This selective pattern of association suggests specificity in the metabolic pathways influenced by dietary live microbes, rather than a general improvement across all aspects of metabolic health. The lack of significant association with FPG contrasts with experimental studies demonstrating improved glycemic control with probiotic supplementation (37). This discrepancy may reflect differences between acute interventional studies and habitual dietary patterns captured in our cross-sectional analysis. It might also indicate that the effects of dietary live microbes on glucose metabolism require higher concentrations, longer exposure periods, or specific microbial strains not typically consumed in standard diets. Similarly, the negligible association with WC suggests that the benefits of dietary live microbes on metabolic health likely operate through mechanisms independent of substantial changes in central adiposity. This discrepancy highlights the complex relationship between dietary microbes and glucose metabolism, which may be influenced by the specific strains present in foods and individual host factors (38). The differential patterns observed across our dual analytical approaches merit consideration. In the categorical analysis, high dietary live microbe intake showed significant associations with lower risk of MetS, HDL-C, TG, and BP. However, in the MedHi continuous analysis, only HDL-C maintained a robust association after full adjustment, with BP showing borderline significance. This suggests that the relationship between dietary live microbes and metabolic outcomes may depend not only on the quantity consumed but also on the specific patterns of consumption, with qualitative differences between food sources potentially playing an important role beyond just the amount consumed.

Our longitudinal analysis revealed perhaps the most clinically relevant finding: the significant reduction in mortality risk among MetS patients with higher dietary live microbe intake. The observed mortality benefit pattern deserves particular attention - the reduction in CVD mortality risk was considerably more pronounced than the more modest reduction in all-cause mortality. This differential impact suggests that dietary live microbes may specifically target cardiovascular pathophysiology beyond their metabolic effects. The stronger protection against CVD death likely reflects the cumulative benefits of improved lipid profiles, better blood pressure control, and potential direct effects on vascular inflammation, endothelial function, and thrombotic pathways not captured in our MetS component analysis (39, 40). This cardiovascular-specific protection aligns with emerging evidence on the gut-heart axis, where microbial metabolites like trimethylamine N-oxide (TMAO) and secondary bile acids directly influence atherosclerotic processes and cardiac function (41). The observed reduction in CVD mortality risk in both medium and high intake groups expands upon work by Guo et al., who reported reduced cardiovascular events in individuals with diabetes consuming foods with medium levels of live microbes (16). Similarly, Liang et al. found that consumption of foods with higher microbial concentrations was associated with reduced CVD-specific mortality in the general population (13), with our study now confirming this benefit specifically in the metabolic syndrome subpopulation. Interestingly, when examining changes in hazard ratios across adjustment models for all-cause mortality, we observed that demographic factors were important negative confounders, strengthening the protective association when adjusted for in Model 2. Conversely, socioeconomic and lifestyle factors appeared to be positive confounders, partially explaining the observed associations when added in Model 3. For CVD mortality, the protective associations were more robust and consistent across all models, suggesting that the relationship between dietary live microbe intake and CVD mortality is less influenced by confounding factors than all-cause mortality. These observations highlight the complex relationship between dietary patterns, demographics, lifestyle factors, and mortality outcomes in individuals with metabolic syndrome.

Foods represent significant sources of ingested microbes that substantially influence gut microbiome composition and diversity (27). This gut microbial ecosystem plays a crucial role in regulating metabolic health and cardiovascular function (42). The observed association between higher dietary live microbe intake and reduced risk of MetS and its components, particularly dyslipidemia and elevated blood pressure, likely involves several interconnected mechanisms. Live microbes contribute to the production of metabolites like short-chain fatty acids (SCFAs), which enhance glucose homeostasis and insulin sensitivity, ultimately modulating blood lipid levels and blood pressure (43). These SCFAs effectively reduce blood lipid levels by inhibiting hepatic lipogenesis and regulating cholesterol metabolism (44), while also influencing blood pressure through effects on vascular tone and renal sodium excretion (45). Dietary live microbes also help attenuate low-grade chronic inflammation, a fundamental pathological mechanism in metabolic syndrome, through their modulation of gut microbiota composition (46), leading to improvements in insulin sensitivity, lipid metabolism, and vascular function. Zhang et al. documented that consuming fermented dairy products with live microbes correlates with lower inflammatory markers and improved metabolic parameters (47).

Our stratified analysis revealed that the relationships between dietary live microbe intake and MetS varied across different subgroups. The most pronounced protective effects were observed in individuals aged 39–59 years, those with above high school education, never smokers, individuals with PIR > 3.5, married/cohabiting individuals, those who were insufficiently active, and never drinkers. Interaction tests revealed significant effect modification by education level and PIR in the relationship between dietary live microbe intake and MetS, suggesting that socioeconomic factors may influence this relationship. These findings may reflect increased opportunities for access to higher quality foods, including more diverse fermented foods and fresh produce, among individuals with higher socioeconomic status (48). While Table 1 shows that a higher percentage of highly educated individuals consume a high microbe diet, the stronger protective association in this group likely reflects multiple factors beyond mere consumption patterns. Higher education may be associated with more health-conscious food preparation methods that preserve microbial viability, better adherence to dietary patterns rich in diverse microbe sources, and potentially synergistic lifestyle factors that enhance the metabolic benefits of dietary live microbes. Additionally, differences in gut microbiome composition and function across socioeconomic strata, as documented in previous studies, may influence how individuals respond metabolically to dietary live microbe intake. More educated individuals may be more aware of the importance of healthy eating and more likely to adopt dietary patterns rich in live microbes (49). These results emphasize the importance of considering socioeconomic factors when developing public health interventions to promote dietary live microbe intake, particularly interventions targeting resource-limited communities.

The main strength of this study lies in its use of a large representative population-based database (NHANES), which allows for generalizability to the broader U.S. population. Additionally, the assessment of both cross-sectional associations and longitudinal survival outcomes enhances our understanding of the role of dietary live microbes in metabolic health and long-term survival. We employed multivariable-adjusted models controlling for a wide range of potential confounders, including demographic, socioeconomic, and lifestyle factors, which strengthens the reliability of our findings. However, this study also has several limitations. First, due to the cross-sectional design of the MetS analysis, we cannot establish causality or temporal sequence between dietary live microbe intake and metabolic syndrome. Second, dietary intake data obtained from 24-h dietary recalls may be subject to recall bias, leading to measurement error. Third, the classification of dietary live microbes was based on expert discussions and literature reviews, lacking precise calculation, which may have introduced some imprecision. Fourth, while the Sanders classification system is one of the most widely accepted methods for assessing dietary live microbes, it may not fully capture the effects of food processing and storage on microbial viability. Furthermore, we did not analyze which specific food groups predominantly drove the observed associations between dietary live microbe intake and health outcomes. Future research should aim to analyze the specific contributions of these individual food categories to better understand their relative importance in the observed health effects and to develop more targeted dietary recommendations.

## 5 Conclusion

In conclusion, this study provides important evidence for associations between dietary live microbe intake and reduced prevalence of metabolic syndrome, as well as improved survival outcomes in individuals with metabolic syndrome. These findings suggest that dietary live microbes may be a promising modifiable factor for the prevention and management of metabolic syndrome and may improve long-term health outcomes through improvements in lipid metabolism, blood pressure control, and attenuation of inflammation. Future research should further explore the causal relationships and potential mechanisms underlying these associations to support the development of specific dietary recommendations for metabolic health.

## Data Availability

Publicly available datasets were analyzed in this study. This data can be found here: all data were extracted from National Health and Nutrition Examination Survey database. The detailed information was provided on the NHANES website (https://www.cdc.gov/nchs/nhanes/).

## Footnotes

^1^ https://www.cdc.gov/nchs/index.html
