# Supplementary material for: Higher dietary live microbe intake is linked to reduced risk of metabolic syndrome and mortality: a cross-sectional and longitudinal study
Source: Front Nutr. 2025 Apr 29;12:1592969. doi: 10.3389/fnut.2025.1592969 (PMC12069296; doi:10.3389/fnut.2025.1592969)
Supplement: Supplementary file 1 [file Table_1.docx]

**Table S1.** Association of dietary live microbe intake with MetS and its components when additionally adjusted for dietary energy intake.

|  | Model 1 |  | Model 2 |  |
| --- | --- | --- | --- | --- |
|  | OR (95%CI) | P value | OR (95%CI) | P value |
| **MetS** |  |  |  |  |
| Low | 1 (Reference) |  | 1 (Reference) |  |
| Medium | 0.95(0.89,1.02) | 0.13 | 0.93(0.86,1.02) | 0.11 |
| High | 0.80(0.73,0.86) | <0.0001 | 0.86(0.77,0.95) | 0.004 |
| P for trend | <0.0001 |  | 0.004 |  |
| **Elevated FPG** |  |  |  |  |
| Low | 1 (Reference) |  | 1 (Reference) |  |
| Medium | 1.03(0.96,1.11) | 0.44 | 1.03(0.94,1.12) | 0.51 |
| High | 0.95(0.86,1.04) | 0.23 | 1.11(1.01,1.23) | 0.03 |
| P for trend | 0.278 |  | 0.038 |  |
| **Low HDL-C** |  |  |  |  |
| Low | 1 (Reference) |  | 1 (Reference) |  |
| Medium | 0.79(0.74,0.84) | <0.0001 | 0.88(0.82,0.95) | <0.001 |
| High | 0.67(0.62,0.72) | <0.0001 | 0.74(0.68,0.81) | <0.0001 |
| P for trend | <0.0001 |  | <0.0001 |  |
| **Elevated TG** |  |  |  |  |
| Low | 1 (Reference) |  | 1 (Reference) |  |
| Medium | 0.96(0.91,1.02) | 0.20 | 0.94(0.88,1.01) | 0.09 |
| High | 0.84(0.78,0.91) | <0.0001 | 0.87(0.79,0.95) | 0.003 |
| P for trend | <0.0001 |  | 0.003 |  |
| **Elevated WC** |  |  |  |  |
| Low | 1 (Reference) |  | 1 (Reference) |  |
| Medium | 0.94(0.89,1.00) | 0.06 | 0.91( 0.82, 1.01) | 0.09 |
| High | 0.87(0.81,0.94) | <0.0001 | 0.91( 0.80, 1.05) | 0.19 |
| P for trend | <0.001 |  | 0.181 |  |
| **Elevated BP** |  |  |  |  |
| Low | 1 (Reference) |  | 1 (Reference) |  |
| Medium | 1.11(1.05,1.19) | <0.001 | 1.01(0.94,1.10) | 0.73 |
| High | 0.88(0.81,0.95) | 0.001 | 0.90(0.82,1.00) | 0.05 |
| P for trend | 0.005 |  | 0.059 |  |

OR, odds ratio; CI, confidence interval; MetS, Metabolic syndrome; FPG, fasting plasma glucose; HDL-C, high-density lipoprotein cholesterol; TG, triglycerides; WC, waist circumference; BP, blood pressure; PIR, poverty income ratio; BMI, body mass index.

Model 1: There are no covariates were adjusted

Model 2: Age, sex, race, education level, marital status, PIR, BMI, smoking status, drinking status, physical activity and dietary energy intake were adjusted.

**Table S2.** Association of dietary MedHi food intake with MetS and its components when additionally adjusted for dietary energy intake.

|  | Model 1 |  | Model 2 |  |
| --- | --- | --- | --- | --- |
|  | OR (95%CI) | P value | OR (95%CI) | P value |
| **MetS** |  |  |  |  |
| MedHi | 0.96(0.94,0.98) | <0.0001 | 0.97(0.94,1.00) | 0.03 |
| MedHi group |  |  |  |  |
| Q1 | 1 (Reference) |  | 1 (Reference) |  |
| Q2 | 0.94(0.86,1.03) | 0.19 | 0.87(0.78,0.98) | 0.02 |
| Q3 | 0.86(0.79,0.95) | 0.002 | 0.88(0.79,0.99) | 0.03 |
| P for trend | 0.002 |  | 0.034 |  |
| **Elevated FPG** |  |  |  |  |
| MedHi | 0.99(0.97,1.01) | 0.40 | 1.00(0.97,1.02) | 0.81 |
| MedHi group |  |  |  |  |
| Q1 | 1 (Reference) |  | 1 (Reference) |  |
| Q2 | 1.00(0.90,1.11) | 0.98 | 0.96(0.85,1.08) | 0.48 |
| Q3 | 0.97(0.88,1.06) | 0.48 | 0.98(0.88,1.09) | 0.67 |
| P for trend | 0.47 |  | 0.682 |  |
| **Low HDL-C** |  |  |  |  |
| MedHi | 0.93(0.90,0.95) | <0.0001 | 0.96(0.94,0.98) | 0.002 |
| MedHi group |  |  |  |  |
| Q1 | 1 (Reference) |  | 1 (Reference) |  |
| Q2 | 0.84(0.77,0.91) | <0.0001 | 0.87(0.79,0.96) | 0.01 |
| Q3 | 0.73(0.67,0.80) | <0.0001 | 0.84(0.76,0.94) | 0.002 |
| P for trend | <0.0001 |  | 0.002 |  |
| **Elevated TG** |  |  |  |  |
| MedHi | 0.97(0.95,1.00) | 0.02 | 0.98(0.96,1.01) | 0.15 |
| MedHi group |  |  |  |  |
| Q1 | 1 (Reference) |  | 1 (Reference) |  |
| Q2 | 0.91(0.84,1.00) | 0.05 | 0.90(0.82,1.00) | 0.04 |
| Q3 | 0.88(0.80,0.96) | 0.004 | 0.89(0.80,0.99) | 0.03 |
| P for trend | 0.004 |  | 0.027 |  |
| **Elevated WC** |  |  |  |  |
| MedHi | 0.95(0.94,0.97) | <0.0001 | 0.99( 0.96, 1.03) | 0.70 |
| MedHi group |  |  |  |  |
| Q1 | 1 (Reference) |  | 1 (Reference) |  |
| Q2 | 0.98(0.91,1.06) | 0.58 | 0.92( 0.81, 1.04) | 0.19 |
| Q3 | 0.85(0.78,0.93) | <0.001 | 0.97( 0.84, 1.12) | 0.64 |
| P for trend | <0.001 |  | 0.677 |  |
| **Elevated BP** |  |  |  |  |
| MedHi | 0.98(0.96,1.00) | 0.007 | 0.98(0.96,1.00) | 0.11 |
| MedHi group |  |  |  |  |
| Q1 | 1 (Reference) |  | 1 (Reference) |  |
| Q2 | 1.11(1.02,1.20) | 0.02 | 0.97(0.88,1.07) | 0.56 |
| Q3 | 1.00(0.92,1.09) | 0.98 | 0.92(0.82,1.02) | 0.12 |
| P for trend | 0.966 |  | 0.115 |  |

MedHi: Medium-high live microbe as continuous variable (in 100 grams); OR, odds ratio; CI, confidence interval; MetS, Metabolic syndrome; FPG, fasting plasma glucose; HDL-C, high-density lipoprotein cholesterol; TG, triglycerides; WC, waist circumference; BP, blood pressure; PIR, poverty income ratio; BMI, body mass index.

Model 1: There are no covariates were adjusted

Model 2: Age, sex, race, education level, marital status, PIR, BMI, smoking status, drinking status, physical activity and dietary energy intake were adjusted.

**Table S3.** Multivariable-adjusted HRs and 95% CIs for dietary live microbe intake in relation to all- cause and CVD Mortality among 12,432 participants with MetS when additionally adjusted for dietary energy intake.

|  | Model 1 |  | Model 3 |  |
| --- | --- | --- | --- | --- |
|  | HR (95%CI) | P value | HR (95%CI) | P value |
| All-cause mortality | | | | |
| Low | 1 (Reference) |  | 1 (Reference) |  |
| Medium | 0.95(0.85,1.07) | 0.43 | 0.89(0.80,0.98) | 0.02 |
| High | 0.80(0.69,0.92) | 0.002 | 0.94(0.81,1.10) | 0.46 |
| CVD mortality | | | | |
| Low | 1 (Reference) |  | 1 (Reference) |  |
| Medium | 0.81(0.68,0.98) | 0.03 | 0.75(0.62,0.91) | 0.003 |
| High | 0.63(0.49,0.81) | <0.001 | 0.76(0.58,0.99) | 0.04 |

HR, hazard ratio; CI, confidence interval; MetS, Metabolic syndrome; CVD, cardiovascular disease; PIR, poverty income ratio; BMI, body mass index.

Model 1: There are no covariates were adjusted

Model 2: Age, sex, race, education level, marital status, PIR, BMI, smoking status, drinking status, physical activity and dietary energy intake were adjusted.

**Table S4.** Multivariable-adjusted HRs and 95% CIs for dietary MedHi food intake in relation to all- cause and CVD mortality among 12,432 participants with MetS when additionally adjusted for dietary energy intake.

|  | Model 1 |  | Model 3 |  |
| --- | --- | --- | --- | --- |
|  | HR (95%CI) | P value | HR (95%CI) | P value |
| All-cause mortality |  |  |  |  |
| MedHi | 0.94(0.90,0.98) | 0.004 | 0.94(0.90,0.98) | 0.01 |
| MedHi group |  |  |  |  |
| Q1 | 1 (Reference) |  | 1 (Reference) |  |
| Q2 | 0.97(0.84,1.12) | 0.70 | 0.89(0.76,1.04) | 0.14 |
| Q3 | 0.89(0.75,1.06) | 0.20 | 0.87(0.73,1.04) | 0.12 |
| CVD mortality |  |  |  |  |
| MedHi | 0.91(0.85,0.99) | 0.002 | 0.91(0.83,1.00) | 0.05 |
| MedHi group |  |  |  |  |
| Q1 | 1 (Reference) |  | 1 (Reference) |  |
| Q2 | 0.93(0.69,1.25) | 0.62 | 0.84(0.62,1.13) | 0.25 |
| Q3 | 0.88(0.65,1.19) | 0.40 | 0.86(0.61,1.20) | 0.36 |

MedHi: medium-high live microbe as continuous variable (in 100 grams); MetS, Metabolic syndrome; HR, hazard ratio; CI, confidence interval; CVD, cardiovascular disease.

Model 1: There are no covariates were adjusted

Model 2: Age, sex, race, education level, marital status, PIR, BMI, smoking status, drinking status, physical activity and dietary energy intake were adjusted.
